# Systematic review of long term follow-up and transitional care in adolescents and adults with esophageal atresia - why is transitional care mandatory?

**DOI:** 10.1007/s00431-023-04893-6

**Published:** 2023-03-11

**Authors:** G. Brooks, M. Gazzaneo, M. Bertozzi, G. Riccipetitoni, A. Raffaele

**Affiliations:** 1 Department of Pediatric Surgery, Fondazione IRCCS Policlinico San Matteo, Pavia, Italy; 2 Department of Clinical, Surgical, Diagnostic and Pediatric Sciences, University of Pavia, Fondazione IRCCS Policlinico San Matteo, Pavia, Italy

**Keywords:** Esophageal atresia, Transitional care, Systematic review, Long term follow-up

## Abstract

Purpose: to review recent literature concerning long-term health issues and transitional care in esophageal atresia (EA) patients. PubMed, Scopus, Embase and Web of Science databases were screened for studies regarding EA patients aged more than or equal to 11 years, published between August 2014 and June 2022. Sixteen studies involving 830 patients were analyzed. Mean age was 27.4 years (range 11–63). EA subtype distribution was: type C (48.8%), A (9.5%), D (1.9%), E (0.5%) and B (0.2%). 55% underwent primary repair, 34.3% delayed repair, 10.5% esophageal substitution. Mean follow-up was 27.2 years (range 11–63). Long-term sequelae were: gastro-esophageal reflux (41.4%), dysphagia (27.6%), esophagitis (12.4%), Barrett esophagus (8.1%), anastomotic stricture (4.8%); persistent cough (8.7%), recurrent infections (4.3%) and chronic respiratory diseases (5.5%). Musculo-skeletal deformities were present in 36 out of 74 reported cases. Reduced weight and height were detected in 13.3% and 6% cases, respectively. Impaired quality of life was reported in 9% of patients; 9.6% had diagnosis or raised risk of mental disorders. 10.3% of adult patients had no care provider. Meta-analysis was conducted on 816 patients. Estimated prevalences are: GERD 42.4%, dysphagia 57.8%, Barrett esophagus 12.4%, respiratory diseases 33.3%, neurological sequelae 11.7%, underweight 19.6%. Heterogeneity was substantial (> 50%).

*Conclusion*: EA patients must continue follow-up beyond childhood, with a defined transitional-care path by a highly specialized multidisciplinary team due to the multiple long-term sequelae.

**Table:** 

**What is Known:** *• Survival rates of esophageal atresia patients is now more than 90% thanks to the improvements in surgical techniques and intensive care, therefore patients’ needs throughout adolescence and adulthood must be taken into account.*	
**What is New:** *• This review, by summarizing recent literature concerning long term sequelae of esophageal atresia, may contribute to raise awareness on the importance of defining standardized protocols of transitional and adulthood care for esophageal atresia patients.*

**Supplementary Information:**

The online version contains supplementary material available at 10.1007/s00431-023-04893-6.

## Introduction

Esophageal atresia (EA) is a rare congenital disorder consisting of the disruption of the esophageal continuity, with or without the persistence of a tracheoesophageal fistula (TEF). The estimated birth incidence is 1:2500–3000 live births with a slight male preponderance [1]. Thanks to the improvement of surgical techniques and intensive care, postnatal survival nowadays is more than 90% [1], meaning that most patients reach adolescence and adulthood. While follow-up throughout childhood is well standardized, the interest in transitional care and its impact on patients’ health is relatively recent and constantly rising. A 2014 meta-analysis [2] quantified the prevalence of long-term issues referable to EA in order to lay the foundations for standardized transitional care protocols. Furthermore, a qualitative study [3] reported physical and mental health problems identified by the patients themselves as relevant and therefore worthy of being addressed in follow-up programs. Nonetheless, shared guidelines for the management of adolescent and adult EA patients are still lacking.

The aim of this study was to review the recent literature concerning long term follow-up and transitional care in adolescent and adult EA patients, in particular to determine whether structured transitional care programs are being followed and their impact on patients’ health.

## Methods

This review was performed according to the PRISMA (Preferred Reporting Items for Systematic Reviews and Meta-Analyses) [4] and SWiM (Synthesis Without Meta-analysis) [5] reporting guidelines.

### Search strategy

PubMed, Embase, Scopus and Web of Science were screened electronically until July 2022, using the key words “transitional care AND esophageal atresia OR long term follow-up AND esophageal atresia”. The search and selection criteria were restricted to the English language. Reference lists of relevant articles and reviews were hand searched for additional reports. The publication span included the period August 2014–June 2022.

### Inclusion criteria

Inclusion criteria were: (1) original studies and (2) sufficient data concerning EA patients aged equal or more than 11 years. The age limit of 11 years old was defined in order to have enough long-term follow-up for outcome evaluation and to avoid including in the analysis younger children. Two authors (GB and MG) reviewed all abstracts independently. Agreement about potential relevance was reached by consensus, and full-text copies of those articles were obtained. Quality assessment was performed by two authors (GB and MG) using the Newcastle–Ottawa Scale [6].

### Exclusion criteria

For the descriptive analysis we excluded: (1) articles published before August 2014 to avoid overlap with the results of another exhaustive meta-analysis that summarized the literature from 1993 to July 2014 [2]; (2) systematic reviews in order to analyze original data; (3) studies that did not report the analyzed outcomes (detailed in the next paragraph) referred to EA patients ≥ 11 years; (4) articles written in languages other than English. For the meta-analysis, we excluded studies with less than 5 patients.

### Data extraction and synthesis

Studies were assessed according to the following variables: population (age at follow-up, years; sex; follow-up, years), clinical characteristics (EA type; associated congenital anomalies), surgical features (type of repair; other surgeries in the first years of life) and outcomes grouped by systems (gastrointestinal, respiratory, musculoskeletal, neurodevelopment, mental health and quality of life, other). The same reviewers mentioned above independently extracted relevant data regarding study characteristics, patients’ features, and outcomes. Inconsistencies were discussed by the reviewers and consensus reached. If more than one study was published for the same cohort with identical end points, the report containing the most comprehensive information on the population was included to avoid overlapping populations. Results are presented with descriptive statistics. Quantitative variables were summarized as mean and ranges, while qualitative variables were summarized as frequency rates. Results for each outcome were calculated considering the subtotal of patients obtained by the studies that reported the outcome. Statistical analysis was performed using Microsoft Office Excel.

Additionally, pooled estimates for the occurrence of main findings were calculated through a meta-analysis that, in order to cope with the presumptive heterogeneity in study design, was designed through a random effect model. I^2^ statistic was then applied in order to estimate the amount of inconsistency between included studies (i.e. the percentage of total variation across studies that could be associated with underlying heterogeneity rather than chance), and the following categorization was taken in account: I^2^ ranging between 0 to 25% = low heterogeneity; I^2^ ranging between 26 and 50% = moderate heterogeneity; I^2^ ≥ 50% = substantial heterogeneity. Publication bias was then investigated through calculation of the contour-enhanced funnel plots, and Egger test for quantitative publication bias analysis (at a 5% of significance level). Radial plots were then calculated and visually inspected to rule out small study bias. All analyses were performed by means of “meta”, “metafor”, and “robvis” packages with R (version 4.0.3) and RStudio (version 1.1.463) software. The aforementioned packages are an open-source add-ons for conducting meta-analyses.

## Results

### Study search and selection

**Fig. 1:**
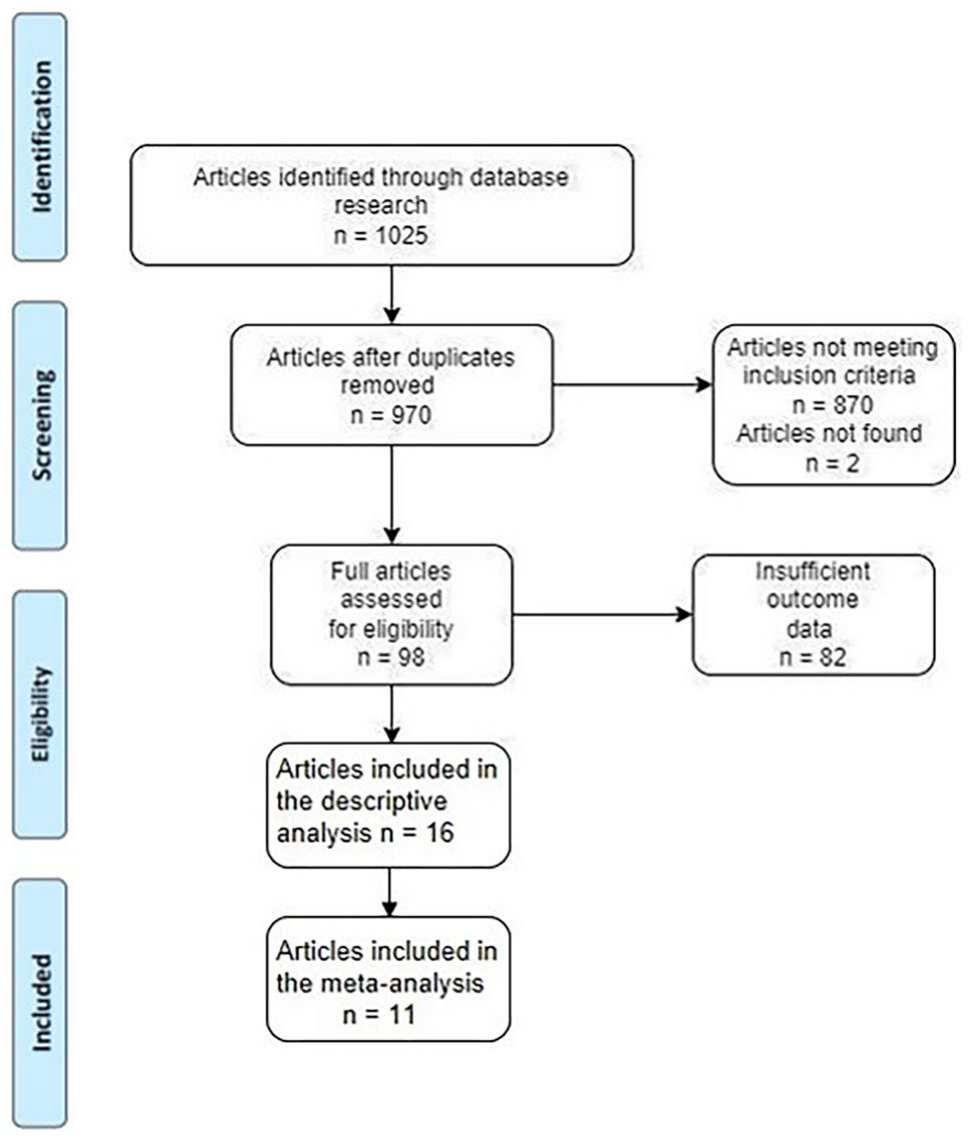
Diagram summarizing the results of the systematic analysis

1025 articles were screened. Excluding duplicates, 16 articles met the inclusion criteria and were considered for descriptive analysis, including 830 patients, while 11 articles met the inclusion criteria for the meta-analysis, with a total of 816 patients (Fig. 1).

### Descriptive analysis

A total of 830 patients were considered in the review. Table 1 [7–22] summarizes the patients’ most important characteristics, as obtained from the analyzed articles.

**Table 1:** Key details of the papers included in the descriptive analysis

**Author**	**n° patients**	**EA type**	**Age at follow-up mean (range)**	**GI outcomes**	**Respiratory outcomes**	**Musculoskeletal outcomes**	**Design**	**Year**
Arneitz et al. [7]	19	A (1); B (1); C (16); D (1)	24.7 y (14–40)	dysphagia (5); GERD (6); other (9)	cough (4); 13 chronic disorder	mean low muscle mass	prospective	2020
Curci and Dibbins [8]	1	C	23 y	GERD	N	scoliosis	case report	2015
Dessanti et al. [9]	2	A (2)	36 y (29–43)	N (2)	n.s.	n.s.	case report	2021
Di Natale et al. [10]	4	A (1); C (3)	23.2 y (n.s.)	dysphagia (3); GERD (1)	n.s.	asymmetric thorax (1); scoliosis (1); rib fusions (1); winged scapula (1)	retrospective	2021
Gatzinsky et al. [11]	29	n.s.	31 y (25–40)	dysphagia (22); GERD (11); esophagitis (14); Barrett (2); other (17)	n.s.	n.s.	retrospective	2016
Gibreel et al. [12]	46	A (4); C (40); D (1); E (1)	40 y (18–63)	dysphagia (38); GERD (12); anastomotic stenosis (18)	n.s.	n.s.	retrospective	2017
Hannon et al. [13]	64	A (14); B (1); C (45); D (4)	29 y (n.s.)	dysphagia (28); GERD (32); anastomotic stenosis (15); Barrett (1); other (20)	chronic disorder (9)	n.s.	retrospective	2019
Hsieh et al. [14]	3	C (3)	15.6 y (13.6–17)	dysphagia (1); Barrett (3); other (1)	n.s.	n.s.	retrospective	2017
Huynh-Trudeau et al. [15]	41	C (35); n.s. (6)	25 y (18–44)	dysphagia (30); GERD (12); esophagitis (8); Barrett (10); other (71)	n.s.	n.s.	prospective	2015
Leibovitch et al. [16]	26	n.s.	n.s. (12–21 y)	GERD (5); anastomotic stenosis (1)	chronic disorder (9); recurrent infections (5); other (2)	n.s.	retrospective	2018
Mikkelsen et al. [17]	68	A (3); C (58); D (4); E (3)	16 y (13–20)	dysphagia (58); GERD (44)	n.s.	n.s..	cross-sectional	2020
Okuyama et al. [18]	69	A (6); C (63)	19 y (15–33)	dysphagia (5); other (3)	oxygen dependence (1); other (2)	scoliosis (25); asymmetric thorax (8); elevated scapula (18)	retrospective	2017
Presse et al. [19]	37	A (6); C (31)	25.3 y (18–44)	dysphagia (24); other (16)	n.s.	n.s	prospective	2016
Schneider et al. [20]	120	A (6); C (108); D (6)	16.5 y (n.s.)	dysphagia (70); GERD (49); esophagitis (80); Barrett (51); anastomotic stenosis (4); other (12)	cough (48)	n.s.	prospective	2016
Svoboda et al. [21]	297	A (34); n.s. (263)	n.s. (≥ 11y)	GERD (169)	recurrent infections (21)	n.s.	retrospective	2018
Vergouwe et al. [22]	4	A (2); C (2)	48.25 y (36–47)	dysphagia (3); GERD (1); esophagitis (1); anastomotic stenosis (2); cancer (5); other (1)	chronic disorder (2)	n.s.	case-series	2018

Esophagitis was defined endoscopically. N° patients per endpoint reported in brackets. EA type as in Gross classification

*y* years, *Y* yes, *N* none, *n.s.* not specified, *GERD* gastro-esophageal reflux disease

Patients’ mean age was 27.4 years (range 11–63 years). Sex was specified in 507 patients: 259 (51%) were male, 248 (49%) female. The most common subtype (according to Gross classification) was type C (405 patients, 48.8%), followed by: type A (79, 9.5%), type D (16, 1.9%), type E (4, 0.5%) and type B (2, 0.2%). In 324 (39%) cases the subtype was not described. The reported associated congenital anomalies were: cardiac in 53 patients (6.4%); gastrointestinal in 28 (3.4%); vertebral in 27 (3.2%); renal in 23 (2.8%); musculoskeletal in 8 (1%); VACTERL/VACTER association in 17 (2%); other than the previously cited in 19 (2.3%). In 472 (57%) cases the presence or absence of other congenital anomalies was not reported. The type of repair was reported in 496 patients: the most common was primary repair (275 patients, 55.4%), followed by delayed repair (170 cases, 34.3%), and esophageal substitution (52 patients, 10.5%). In the latter subgroup, the transposition performed was gastric in 37 cases, colonic in 5 and jejunal in 2; the transposed organ was not specified in 8 cases. Concerning other surgeries during childhood, esophageal dilatations are reported in 142 patients (17%), with a range of 1–108 dilatations per patient [17]; fundoplication in 105 (12.6%); aortopexy in 10 (1.2%); repair of recurrent TEF in 5 (0.6%); other not specified surgeries in 5 (0.6%). Mean age at follow-up was 27.4 years (range 11–63 years).

All studies reported data concerning gastrointestinal outcomes. One hundred sixty one patients [15, 20] were evaluated for the study purposes (either through endoscopy, barium swallow and manometry), while in the remaining 669 cases data was obtained retrospectively. The most frequently reported signs were GERD symptoms (gastro-esophageal reflux disease, 344 patients, 41.4% of total) and dysphagia (229 cases, 27.6%). A hiatal hernia was identified in 33 cases (4%), while esophagitis was histologically documented in 103 patients (12.4%) and Barrett esophagus in 67 (8.1%). Anastomotic stricture was present in 40 patients (4.4%) and food blockage was observed in 22 (2.6%) cases. Esophageal hypomotility was documented in 55 patients (6.6%). 10 patients (1.2%) necessitated nutritional support. 5 (0.6%) cases of esophageal cancer were reported. Other symptoms were signaled in 30 patients (3.6%), such as upper gastrointestinal bleeding, dumping syndrome and the necessity of revision surgery [13].

Respiratory outcomes were reported in 600 out of 830 patients. Nineteen patients underwent prospective clinical examination, spirometry and spiroergometry [7]. Persistent cough was described in 52 cases (8.7%); chronic respiratory disease (not specified whether obstructive or restrictive) in 33 (5.5%); recurrent respiratory infections in 26 (4.3%); asthma in 10 (1.7%); restrictive respiratory disorder in 9 (1.5%); other morbidities like oxygen-dependence and recurrent TEF [18] in 8 (1.3%). A study found that EA patients present minor alterations in respiratory microbiome as compared to healthy peers, associated with decreased performance capacity and peak VO2 (volume of oxygen consumed) [7].

Musculoskeletal outcomes were described in 74 patients. Recent X-ray examinations were reviewed in 4 cases [10]. Scoliosis was observed in 27 patients (36.5%); winged or elevated scapula in 19 (25.7%); chest wall deformity in 9 (12%); rib fusion in 1 (1.3%). A study pointed out a lower mean muscle mass compared to controls [7].

Neurological sequelae were delineated in 135 patients: there are 12 reported cases of mental retardation (8.9%) and 1 of cerebral palsy (0.7%).

Mental health and quality of life (QoL) were evaluated in 5 studies, totaling 208 patients; a reduced QoL compared to healthy peers was identified using validated questionnaires in 19 cases (9%). Mental disorders were present in 20 patients (9.6%): depression in 9, PTSD (post-traumatic stress disorder) in 6 and a raised risk to develop an overt mental illness in 5 [17].

Among other long term outcomes observed (700 patients), 93 patients (13.3%) were underweight and 42 (6%) had a reduced height compared to general population, while only 13 patients were obese (1.8%). 3 patients (0.4%) underwent a renal transplant in adulthood. Chronic anemia was reported in 18 cases (2.6%), 15 of whom were corrected through gastric transposition. Allergy or food intolerance were described in 9 patients (1.3%). Interestingly, 72 patients (10.3%) had no care provider [21].

### Meta-analysis

**Fig. 2:**
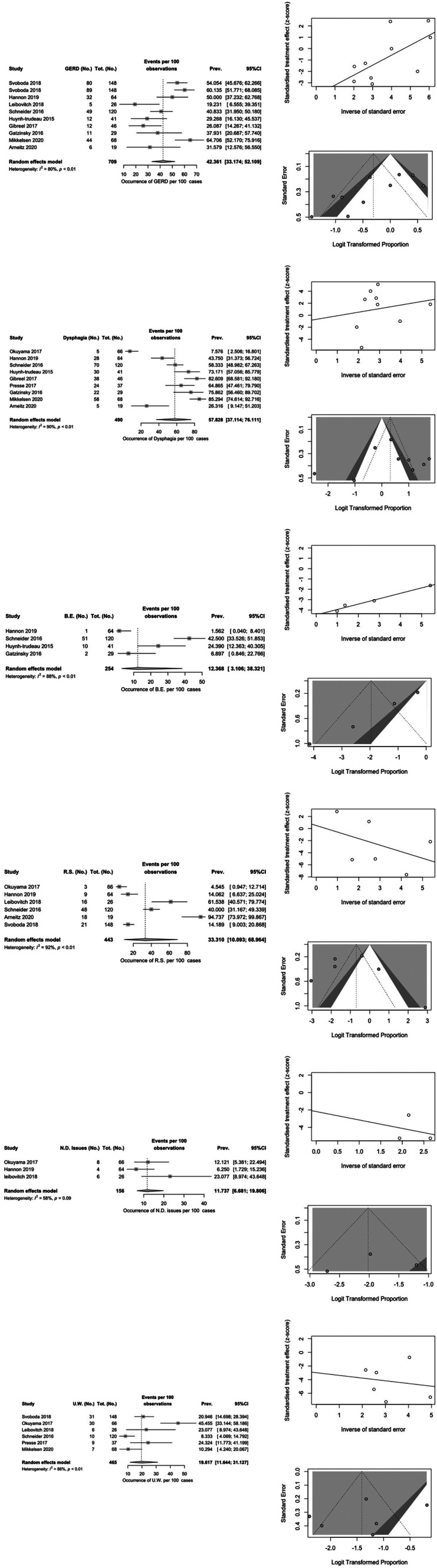
Forest plots of pooled estimated prevalence alongside with the related funnel plots and standard error

Eleven studies with more than 5 patients were considered for meta-analysis after quality assessment with New-Ottawa scale (Table 2). A total of 816 patients were considered. Figure 2 shows the forest and funnel plots for GERD, dysphagia, Barrett esophagus, respiratory sequelae, neurological sequelae and underweight.

Ten studies reported data on GERD (340 patients). The estimated prevalence is 42.4%, with a 95% CI (confidence interval) of 33.2% to 52.1% and heterogeneity of 80%.

**Table 2:** Quality Assessment [6]

**Author**	**Year**	**Country**	**Quality Score**	**Ottawa Score**
Arneitz et al. [7]	2020	Austria	high quality	7
Gatzinsky et al. [11]	2016	Sweden	high quality	7
Gibreel et al. [12]	2017	USA	high quality	7
Hannon et al. [13]	2019	UK	high quality	7
Huynh-Trudeau et al. [15]	2015	Canada	high quality	7
Leibovitch et al. [16]	2018	Israel	high risk	6
Mikkelsen et al. [17]	2020	Norway	high quality	7
Okuyama et al. [18]	2017	Japan	high quality	7
Presse et al. [19]	2016	Canada	high quality	7
Schneider et al. [20]	2016	France, Canada, Belgium	high quality	7
Svoboda et al. [21]	2018	Germany, UK	high risk	6

Dysphagia was reported in 9 studies (280 patients), with an estimated prevalence of 57.8% (95% CI 37.1% to 76.1%) and heterogeneity of 90%.

Barrett esophagus was described in 4 studies (64 patients). The estimated prevalence is 12.4% (95% CI 3.1% to 38.3%) and heterogeneity of 88%.

Overall, respiratory sequelae (cough, recurrent respiratory infections, chronic respiratory disorders) were reported in 6 studies (115 patients), with an estimated prevalence of 33.3% (95% CI 10.1% to 69%) and heterogeneity of 92%.

Neurological sequelae were described in 3 studies (18 patients). Estimated prevalence is 11.7% (95% CI 6.7% to 19.8%), with a heterogeneity of 58%.

Lastly, 6 studies reported 93 underweight patients, with an estimated prevalence of 19.6% (95% CI 11.6% to 31.1%) and heterogeneity of 86%.

Details of the meta-analysis can be found in the Supplementary file online.

## Discussion

Advances in surgical techniques and intensive care have resulted in a radical improvement in the survival of patients with congenital anomalies as EA. At the same time, these advances present new challenges for the pediatric surgeon, by creating a subset of adult patients with characteristic long-term outcomes and specific needs. Transitional care has been defined as a planned movement of adolescents and young adults with chronic physical and medical conditions from child-centered to adult-oriented health-care systems [23]. It is gaining more and more importance in the management of EA patients; efforts must be put in creating standardized follow-up protocols throughout adolescence and adulthood to avoid and promptly treat detrimental consequences on patients’ health [24]. Positive impact of transitional care has been reported in a 2017 study [25], in which a two-day educational program for EA adolescents and their parents met a high satisfaction, but at the same time highlighted a low pre-existing transition-specific knowledge. The aim of this systematic review is to underline the long-term health problems in EA patients in order to establish proper follow-up and transitional care protocols to be shared with adult specialists: gastroenterologists, surgeons, pneumologists, orthopedics, neurologists and psychologists.

The analyzed sample includes 830 adolescent and adult EA patients’, aging from 11 to 63 years. Findings concerning long term outcomes were consistent with other reports in literature, in particular with a recent meta-analysis by Connor et al. [2], as gastrointestinal symptoms are the most reported complaint, the most frequent being GERD (41.4%) and dysphagia (27.6%). These and other symptoms, such as food blockage due to anastomotic stenosis or esophageal hypomotility, have a detrimental impact on quality of life. Moreover, the reported prevalence of esophagitis (12.4%), Barrett esophagus (8.1%) and esophageal cancer (0.6%) renders standardized endoscopic follow-up mandatory. A consensus conference conducted by ERNICA (European Reference Network for Rare Inherited Congenital Anomalies) [26] aimed to define surgical follow-up guidelines for EA patients, reviewing simultaneously the ESPGHAN (European Society for Pediatric Gastroenterology Hepatology and Nutrition) 2016 guidelines [27]. General consensus was reached regarding the necessity of a scheduled endoscopic and 24-ph or ph-impedance monitoring in EA children and adolescents, although the frequency of such exams was not defined; on the other hand, an agreement concerning the role of routine contrast upper gastrointestinal studies was not reached. Concerning adult patients, the ESPGHAN surveillance guidelines were considered adequate: routine endoscopy (with biopsies in four quadrants at gastroesophageal junction and anastomotic site) at time of transition into adulthood and every 5–10 years; on-demand adjunctive endoscopies according to the presence of new or worsening symptoms or of Barrett esophagus. Another recent study [28] also suggested following the ESPGHAN guidelines to tackle adequately gastrointestinal issues in EA adult patients.

Respiratory symptoms have been underreported compared to digestive ones, being specified in 600 out of 830 patients. The most common were persistent cough (8.7%); chronic respiratory diseases (8.7%) and recurrent respiratory infections (4.3%); our data were not sufficient to determine the correlation with GERD. Respiratory symptoms reported in EA children can persist in adulthood, as already highlighted in previous studies [29]. The etiopathogenesis appears to be multifactorial [30]: the malformation itself, particularly if a TEF was present; persistent GERD; an acquired damage after surgery and repeated respiratory infections in childhood. Nonetheless, respiratory symptoms are often overlooked by general practitioners and patients’ themselves compared to digestive ones [18]. Therefore, EA patients’ pulmonary function should be regularly assessed, particularly in patients who underwent esophageal substitution as they may present, additionally, a reduced lung capacity due to compression by the transposed organ [13]. During the ERNICA conference [26], general consensus was reached regarding the necessity of scheduled lung function tests in EA children and adolescents, while routine bronchoscopies are not deemed necessary. A recent paper endorsed by INoEA (International Network of Esophageal Atresia) also suggests the use of pulmonary function tests in the follow-up of adolescent EA patients, along with chest CT scan for detection of bronchiectasis [31]. We did not find specific protocols for a pneumological follow-up in EA adult patients in literature.

The musculoskeletal morbidities described appear consistent with those thoroughly reported in literature [32]. Although our data do not allow us to correlate these outcomes with the presence of vertebral congenital anomalies nor with the surgical technique, they seem more common in patients who underwent a thoracotomy [18]. In the cohort reported by Di Natale et al. [10], rib fusions and winged scapula were reported more frequently in subjects aged above 18 years old than in other age groups; probably in this group thoracoscopy or muscle-sparing thoracotomy were performed less often. In a recent systematic review [33], secondary scoliosis after thoracotomy repair in EA children is reported in 13% of cases; according to a meta-analysis by Drevin et al. [34], musculoskeletal complications are less common with the thoracoscopic approach. Considering only adult patients, a study by Sistonen et al. [35] reported a 13-fold risk of scoliosis compared to the general population, with rib fusions post-thoracotomy being the strongest predictive factor; however, spinal surgery was never needed. As further studies are needed to define technique-related differences in musculoskeletal outcomes, efforts must be put also in defining the orthopedic follow-up in adult EA patients.

Considering neurological outcomes, 8.9% of patients were diagnosed with mental retardation; it was not possible to define the correlation with prematurity, associated congenital anomalies or other perinatal factors. Impairment in motor and cognitive function in EA children are well described in literature [36]. Early identification is essential in these patients to limit detrimental effects through supportive services such as occupational therapy, speech and language therapy, dietetics and physiotherapy teams [37]. Nonetheless, we did not find studies reporting details concerning neurological follow-up in adolescence and adulthood.

According to our data, 9% of patients reported a reduced QoL and 9.6% presented a mental disorder or a raised risk to develop one. An adequate transitional care program, including psychiatric and psychological support, can prevent development and worsening of such conditions, whereas the absence of such programs cause patients to feel “thrown into the unknown” [38], negatively influencing QoL and mental health.

Interestingly, a reduced weight (13.3%) and height (6%) can persist in adult EA patients. A recent study [39], highlighted risk factors for poor nutrition in EA children and the importance of having an early personalized nutritional program to prevent undergrowth. Our data suggest including nutritional consultations when planning a transitional care program.

Lastly, 10.3% adult EA patients reported to have no care provider [21]; this data underlines that current efforts in improving transitional care must be continued and implemented. In particular, as promoted by ERNICA [26], centralization of rare congenital diseases can improve patient care in every life phase, from birth and throughout adulthood, by registering cases and offering standardized and up-to-date healthcare.

The meta-analysis allowed us to define estimated prevalence for major outcomes (GERD, dysphagia, Barrett esophagus, respiratory sequelae, neurological sequelae and underweight), confirming that a significant proportion of EA patients is affected by long term health problems with a potentially high impact on quality of life. The most significant outcome appears to be dysphagia, with an estimated prevalence of 57.8%. However, due to the substantial heterogeneity of the included studies, no conclusions concerning the effective impact of these conditions can be drawn. Prospective transitional care programs can be useful to define the prevalence of long-term outcomes and how they can be reduced with adequate follow-up.

## Conclusions

### Limitations

Limitations and bias derive from the features of the studies included in this review: most studies are retrospective studies lacking some of the target data, therefore a complete evaluation of all long-term outcomes was not possible and a more extensive meta-analysis could not be performed. Furthermore, no study reported transitional care programs and a long-term evaluation of their impact on patients’ health.

### Implications for clinical practice and research

It is well known that numerous long-term outcomes can persist in EA patients throughout adolescence and adulthood. Patients with congenital malformations seek for information concerning transfer to adult care and require a regular follow-up by specialized centers [40]. Although both EA long term outcomes and patient-driven proposals for follow-up programs are well described in literature, further studies reporting transitional-care programs and their effects on patients’ health are needed in order to help to improve current care practices for adolescent and adult EA patients. As a matter of fact, in clinical practice adult EA patients (as well as patients with other congenital malformations) often can rely only on pediatric specialists for regular follow-up, as knowledge regarding these patients is still limited among general surgeons and practitioners. This issue derives from the fact that congenital malformations are rare diseases and therefore uncommonly encountered in adult care practice. We hope that this review, which summarizes recent literature concerning adolescent and adult EA patients, can raise awareness about these patients’ needs among not only pediatricians and pediatric surgeons but also adult care providers. The ultimate goal is to improve transitional care by standardizing protocols in specialized centers as well as keeping general practitioners, adult specialists and general surgeons updated on this subset of patients that will be more and more encountered in everyday practice. The adoption of prospective standardized protocols in the early treatment, follow-up and transitional care of EA patients is an important and attractive field of clinical research, with positive effects on clinical practice and on the outcomes of this complex malformation.

## Supplementary Information

Below is the link to the electronic supplementary material.

Supplementary file1 (DOCX 16 KB)

## Data Availability

The datasets generated during and/or analysed during the current study are available from the corresponding author on reasonable request.
