# Supplementary material for: Systematic review of long term follow-up and transitional care in adolescents and adults with esophageal atresia - why is transitional care mandatory?
Source: Eur J Pediatr. 2023 Mar 11;182(5):2057–66. doi: 10.1007/s00431-023-04893-6 (PMC10175361; doi:10.1007/s00431-023-04893-6)
Supplement: Supplementary file 1 — Supplementary file1 (DOCX 16 KB) [file 431_2023_4893_MOESM1_ESM.docx]

Review: Pooled Prevalence of Barrett's Esophagus

events 95%-CI

Hannon 2019 1.5625 [ 0.0396; 8.4010]

Schneider 2016 42.5000 [33.5256; 51.8535]

Huynh-trudeau 2015 24.3902 [12.3632; 40.3046]

Gatzinsky 2016 6.8966 [ 0.8464; 22.7662]

Number of studies combined: k = 4

Number of observations: o = 254

Number of events: e = 64

events 95%-CI

Random effects model 12.3677 [3.1064; 38.3208]

Quantifying heterogeneity:

tau^2 = 1.9053; tau = 1.3803; I^2 = 87.7% [70.8%; 94.8%]; H = 2.85 [1.85; 4.40]

Test of heterogeneity:

Q d.f. p-value Test

24.40 3 < 0.0001 Wald-type

52.70 3 < 0.0001 Likelihood-Ratio

Review: Pooled Prevalence of Dysphagia

events 95%-CI

Okuyama 2017 7.5758 [ 2.5056; 16.8007]

Hannon 2019 43.7500 [31.3735; 56.7241]

Schneider 2016 58.3333 [48.9822; 67.2632]

Huynh-trudeau 2015 73.1707 [57.0555; 85.7787]

Gibreel 2017 82.6087 [68.5809; 92.1797]

Presse 2017 64.8649 [47.4611; 79.7900]

Gatzinsky 2016 75.8621 [56.4600; 89.7016]

Mikkelsen 2020 85.2941 [74.6137; 92.7160]

Arneitz 2020 26.3158 [ 9.1466; 51.2029]

Number of studies combined: k = 9

Number of observations: o = 490

Number of events: e = 280

events 95%-CI

Random effects model 57.8280 [37.1138; 76.1109]

Quantifying heterogeneity:

tau^2 = 1.5300; tau = 1.2369; I^2 = 90.5% [84.2%; 94.3%]; H = 3.24 [2.52; 4.18]

Test of heterogeneity:

Q d.f. p-value Test

84.07 8 < 0.0001 Wald-type

134.20 8 < 0.0001 Likelihood-Ratio

Review: Pooled Prevalence of Barrett's Esophagus

events 95%-CI

Hannon 2019 1.5625 [ 0.0396; 8.4010]

Schneider 2016 42.5000 [33.5256; 51.8535]

Huynh-trudeau 2015 24.3902 [12.3632; 40.3046]

Gatzinsky 2016 6.8966 [ 0.8464; 22.7662]

Number of studies combined: k = 4

Number of observations: o = 254

Number of events: e = 64

events 95%-CI

Random effects model 12.3677 [3.1064; 38.3208]

Quantifying heterogeneity:

tau^2 = 1.9053; tau = 1.3803; I^2 = 87.7% [70.8%; 94.8%]; H = 2.85 [1.85; 4.40]

Test of heterogeneity:

Q d.f. p-value Test

24.40 3 < 0.0001 Wald-type

52.70 3 < 0.0001 Likelihood-Ratio

Review: Pooled Prevalence of Neurodevelopmental Issues

events 95%-CI

Okuyama 2017 12.1212 [5.3811; 22.4940]

Hannon 2019 6.2500 [1.7290; 15.2363]

leibovitch 2018 23.0769 [8.9740; 43.6475]

Number of studies combined: k = 3

Number of observations: o = 156

Number of events: e = 18

events 95%-CI

Random effects model 11.7365 [6.6808; 19.8062]

Quantifying heterogeneity:

tau^2 = 0.0997; tau = 0.3157; I^2 = 57.6% [0.0%; 87.9%]; H = 1.54 [1.00; 2.88]

Test of heterogeneity:

Q d.f. p-value Test

4.72 2 0.0944 Wald-type

4.81 2 0.0902 Likelihood-Ratio

Review: Pooled Prevalence of Respiratory Symptoms

events 95%-CI

Okuyama 2017 4.5455 [ 0.9474; 12.7143]

Hannon 2019 14.0625 [ 6.6374; 25.0237]

Leibovitch 2018 61.5385 [40.5708; 79.7740]

Schneider 2016 40.0000 [31.1668; 49.3389]

Arneitz 2020 94.7368 [73.9719; 99.8668]

Svoboda 2018 14.1892 [ 9.0029; 20.8684]

Number of studies combined: k = 6

Number of observations: o = 443

Number of events: e = 115

events 95%-CI

Random effects model 33.3095 [10.0934; 68.9643]

Quantifying heterogeneity:

tau^2 = 3.2521; tau = 1.8034; I^2 = 92.4% [86.3%; 95.8%]; H = 3.64 [2.70; 4.90]

Test of heterogeneity:

Q d.f. p-value Test

66.21 5 < 0.0001 Wald-type

106.08 5 < 0.0001 Likelihood-Ratio

Review: Pooled Prevalence of Underweight Status

events 95%-CI

Svoboda 2018 20.9459 [14.6975; 28.3940]

Okuyama 2017 45.4545 [33.1439; 58.1863]

Leibovitch 2018 23.0769 [ 8.9740; 43.6475]

Schneider 2016 8.3333 [ 4.0686; 14.7915]

Presse 2017 24.3243 [11.7725; 41.1992]

Mikkelsen 2020 10.2941 [ 4.2400; 20.0665]

Number of studies combined: k = 6

Number of observations: o = 465

Number of events: e = 93

events 95%-CI

Random effects model 19.6173 [11.6439; 31.1272]

Quantifying heterogeneity:

tau^2 = 0.4764; tau = 0.6902; I^2 = 86.2% [72.1%; 93.2%]; H = 2.69 [1.89; 3.83]

Test of heterogeneity:

Q d.f. p-value Test

36.24 5 < 0.0001 Wald-type

39.44 5 < 0.0001 Likelihood-Ratio

Review: Pooled Prevalence of Dysphagia

Linear regression test of funnel plot asymmetry

Test result: t = -0.19, df = 7, p-value = 0.8537

Sample estimates:

bias se.bias intercept se.intercept

-0.6862 3.5870 0.5949 1.1333

Details:

- multiplicative residual heterogeneity variance (tau^2 = 11.9480)

Review: Pooled Prevalence of GERD

Linear regression test of funnel plot asymmetry

Test result: t = -2.75, df = 8, p-value = 0.0251

Sample estimates:

bias se.bias intercept se.intercept

-4.0779 1.4840 0.8762 0.3652

Details:

- multiplicative residual heterogeneity variance (tau^2 = 2.8762)

Review: Pooled Prevalence of Neurodevelopmental Issues

Linear regression test of funnel plot asymmetry

Test result: t = -0.24, df = 1, p-value = 0.8519

Sample estimates:

bias se.bias intercept se.intercept

-2.1846 9.2220 -0.9693 4.0708

Details:

- multiplicative residual heterogeneity variance (tau^2 = 4.4700)

Review: Pooled Prevalence of Respiratory Symptoms

Linear regression test of funnel plot asymmetry

Test result: t = 0.14, df = 4, p-value = 0.8984

Sample estimates:

bias se.bias intercept se.intercept

0.4974 3.6568 -1.0837 1.1151

Details:

- multiplicative residual heterogeneity variance (tau^2 = 16.4774)

Review: Pooled Prevalence of Underweight Status

Linear regression test of funnel plot asymmetry

Test result: t = -0.76, df = 4, p-value = 0.4918

Sample estimates:

bias se.bias intercept se.intercept

-2.9840 3.9477 -0.3962 1.1749

Details:

- multiplicative residual heterogeneity variance (tau^2 = 7.9271)
